# Not here yet, but one bite away: Risk for vector-borne zoonotic diseases

**DOI:** 10.1371/journal.pntd.0013802

**Published:** 2025-12-15

**Authors:** Didot Budi Prasetyo, Bruno Mathieu, Jérôme Depaquit, Sébastien Boyer

**Affiliations:** 1 Medical and Veterinary Entomology Unit, Institut Pasteur du Cambodge, Phnom Penh, Cambodia; 2 Faculté de Pharmacie, Université de Reims Champagne Ardenne, UR ESCAPE-USC ANSES PETARD, Reims Cedex, France; 3 Institutes of Bacteriology and Parasitology, Medical Faculty, University of Strasbourg, Strasbourg, France; 4 Pôle de Biologie Territoriale, Laboratoire de Parasitologie-Mycologie, Centre Hospitalo-Universitaire, Reims, France; 5 Ecology & Emergence of Arthropod-Borne Pathogens Unit, Department of Global Health, Institut Pasteur, Paris, France; Makerere University, UGANDA

## Abstract

Recent data demonstrate that vector-borne diseases (VBDs) are expanding into new regions across the globe. This trend is intensified by the interconnected world characterized by rapid urbanization, environmental changes, and increased human mobility, all of which create favorable conditions for the spread of both vectors and pathogens. Despite this growing threat, many countries still rely on reactive surveillance strategies, responding only after outbreaks occur. This approach focuses mainly on well-known vector species, while other potentially important or emerging vectors remain overlooked. Proactive vector surveillance is essential to anticipate and potentially prevent future outbreaks and monitor the importation of pathogens. The authors call on relevant authorities, researchers, and stakeholders to expand and strengthen vector surveillance systems, with particular emphasis on the inclusion of lesser-known vector species.

## Introduction

Vector-borne zoonotic diseases (VBDs) are among the most dynamic and complex global public health threats costing billions of US dollars annually [1]. While some regions may appear unaffected today, recent history has shown how the introduction of a single infected vector or human can lead to widespread outbreaks [2,3]. In a globalized world defined by rapid urbanization, ecological change, and increasing human mobility, the line between safety and epidemic is thinner than ever; sometimes, a single bite can bring a real threat: numerous examples emphasize this precarious balance. The 2007 Chikungunya outbreak in Italy [2], initiated by an infected traveler and sustained by local *Aedes albopictus* population, led to the virus’s unexpected autochthonous transmission in a temperate European country. Similarly, the emergence of Zika virus in Micronesia [4] and the establishment of dengue in metropolitan France [5] demonstrate how a single introduction, followed by efficient vector transmission, can lead to disease endemicity. These examples highlight a critical gap in public health policy: surveillance systems must be more proactive rather than reactive. While this viewpoint focuses primarily on VBDs that pose direct risks to human health, we acknowledge that many plant vector-borne diseases, whether caused by bacteria or viruses and transmitted by various arthropods ranging from aphids, planthoppers, to mites, likewise have major public health implications through their impact on food security [6].

## Does today’s reactive approach to neglected vectors contribute to future outbreaks?

The expansion trends have been observed among vector-borne diseases beyond those transmitted by mosquitoes. The 2006 bluetongue virus (BTV) outbreak illustrates how an unexpected introduction of a virus can lead to its rapid spread across Europe in under a year, by an unsuspected indigenous species of *Culicoides* biting midges [7]. History is repeating itself with the emergence of novel BTV serotype-3 in 2022, which spread through the same route [8]. Another recent example is the re-emergence of Oropouche virus, also transmitted by *Culicoides*, in Brazil during 2023–2024, with evidence of travel-associated spread to other Latin American countries and fears of transmission to another continent [9]. Pathogenic novel tick-borne viruses from the genus Orthonairovirus have also recently been detected in China in both human and tick samples, highlighting their public health importance [10]. Sand fly-borne diseases such as leishmaniasis, long considered as imported in Southeast Asia, have transitioned to locally transmitted cases, particularly in Thailand [11]. These growing examples are alarming, since the vectors and their associated diseases are most often neglected in both surveillance and response efforts. Neglected or less well-known vectors include *Culicoides* spp., Phlebotomine sand flies (mainly genus *Phlebotomus*, *Lutzomyia*, *Nyssomyia*, and *Psychodopygus* spp.), blackflies (*Simulium* spp.), Tse-tse flies (*Glossina* spp.), fleas, lice, bugs, as well as synanthropic insects such as cockroaches and houseflies, which can act as mechanical carriers of pathogens. The neglect of these vectors creates blind spots in global health diagnosis. Effective Integrated Vector Management approaches depend on robust surveillance of all potential vectors, not just those currently associated with outbreaks. As demonstrated by the recent examples of VBDs expansion, today’s neglected species may be tomorrow’s dominant vectors.

## Why is it more relevant now?

The threat is especially relevant now, given the intensifying pace of globalization. International travel is easier than ever, aided by a more open border policies and regional labor migration. For example, within Southeast Asia alone, there are an estimated 20.2 million migrants, nearly 6.9 million of whom have moved within the region [12]. This level of human mobility increases the likelihood of asymptomatic carriers introducing pathogens into areas with suitable vectors. Human population expansion into new areas (often involving deforestation) has increased human–vector–wildlife contact, creating additional opportunities for spillover of zoonotic diseases [13]. In addition, warming temperatures, altered rainfall patterns, and extreme weather events are reshaping the geographical distribution and seasonal activity of vectors [14,15], expanding the range of many species into previously unsuitable areas.

Despite initiatives, such as the WHO Arbovirus Surveillance Network, the European VectorNet or CLIMOS projects, or national dengue and malaria programs, the surveillance remain pathogen-centered and reactive; most lacking standardized cross-border data-sharing and early-warning mechanisms. Finally, armed conflicts and natural disasters also contribute to the VBDs risk by disrupting vector control program and health service, displacing populations of refugee into temporary settlement, often with inadequate sanitation, which have historically fueled outbreaks of VBDs [16].

## Need for proactive vector surveillance

Vector surveillance generally receives limited resources, particularly in low-income regions facing the heaviest VBD burden [17], and the situation is even worse for understudied and neglected vector species. Nonetheless, surveillance of neglected vectors is crucial for preventing and controlling VBDs, particularly in areas where ecological transitions are happening [18]. Effective vector surveillance involves regular collection and analysis of data on vector populations and their distribution. The key elements of successful surveillance include the monitoring of vector populations with regular tracking of species abundance using standardized sampling method, identification and mapping of breeding habitats, the assessment of appropriate control measures, and the development of early-warning systems in regions with elevated risk. Though all of these efforts must be supported by skilled and well-trained medical entomologists, the lack of academic curricula and expertise in fieldwork and vector taxonomy, despite urgent need, has repeatedly been raised as a challenge in some countries [19]. Finally, incorporating pathogen detection in vectors, despite the cost, could reveal silent transmission chains and identify potential outbreak foci. Though efforts have been made to strengthen vector surveillance systems [17,20], current surveillance strategies still face several practical challenges that need to be addressed, which are summarized in Table 1.

**Table 1:** Current vector surveillance remains insufficient due to several limitations.

Current state	Issues	Potential Solutions
Reactive rather than proactive	Surveillance often initiated only after outbreaks occur	Establish continuous sentinel surveillance; strengthen early warning systems
Narrow focus on single vector	Concentration on one species while overlooking other epidemiologically important taxa	Expand taxonomic scope; integrate surveillance of multiple vectors and pathogens
Lack of technical capacity for novel/innovative tools	Limited ability at local level to apply novel/innovative methods [21]	Invest in academic partnerships, training, and technology transfer; capacity building in endemic regions
Poor multisectoral coordination	Absence of multidisciplinary approaches and weak collaboration across sectors [22]	Promote One Health approaches; establish inter-sectoral task forces
Neglected areas excluded	Failure to reach certain location and other underserved areas [23]	Community engagement, citizen science, and participatory surveillance
Lack of continuous funding	Reliance on short-term or emergency-based support or funding fluctuation [17]	Advocate for sustainable financing; embed surveillance in national health budgets

## Anticipating the next vector-borne disease

Anticipating the emergence of new vector-borne pathogens can begin with important yet achievable first steps: raising awareness among policymakers, researchers, and the public, especially regarding lesser-known vectors and their associated diseases. This can include simple yet effective activities such as social media campaigns and targeted outreach to enhance knowledge and encourage community participation [24]. Community can contribute data through passive surveillance and broaden the geographic reach of vector monitoring activity. When integrated with appropriate system such as mobile phone application and crowd-source reporting platform, community participations can strengthen early detection of emerging vector threat [25,26].

More complex but essential next steps include investing in medical entomology curricula, obtaining technical support, and promoting knowledge exchange, which can be facilitated through regional networks and bilateral collaborations with other countries. These activities ensure the standardized monitoring procedures are applied across areas and data are interpreted by skilled personnel. Academic institutions play a central role by training future entomologist, advancing research, and providing the technical foundation. Partnerships with government agencies and international stakeholders open new funding opportunities and enable the translation of entomological findings into policy recommendations. When coordinated across local, national, and regional levels, such a framework facilitates early detection of emerging threats and supports scalable interventions with real-world impact.

## Conclusion

Current vector control strategies are pathogen-centered and remain reactive. Effective prevention demands a shift toward proactive, vector-centered surveillance, with the inclusion of neglected vector species. This approach can help countries gather critical information, anticipate outbreaks, and alert populations to emerging arboviral threats in a rapidly changing world. Ignoring these vectors today may lead to tomorrow’s outbreaks. Preparedness must begin before the bite: the next epidemic is not here yet, but it may be just one bite away.
